# Prosocial Behaviour, Individualism, and Future Orientation of Chinese Youth: The Role of Identity Status as a Moderator

**DOI:** 10.3390/bs15020193

**Published:** 2025-02-11

**Authors:** Raymond Chi-fai Chui, Hang Li, Chi-keung Chan, Nicolson Yat-fan Siu, Raysen Wai-leung Cheung, Wang-on Li, Kelly Zheng-min Peng, Yuet-Wah Cheung, Siu-fung Cheung, Naizan Xu

**Affiliations:** 1 Department of Social Work, Hong Kong Shue Yan University, Hong Kong; 2 Department of Sociology, The Chinese University of Hong Kong, Hong Kong; 3 School of Arts and Humanities, Tung Wah College, Hong Kong; 4 Division of Social Science, The Hong Kong University of Science and Technology, Hong Kong; 5 Department of Counselling and Psychology, Hong Kong Shue Yan University, Hong Kong; 6 Department of Hospitality and Business Management, Technological and Higher Education Institute of Hong Kong, Hong Kong; 7 Department of Sociology, Hong Kong Shue Yan University, Hong Kong; 8 Independent Researcher, Hong Kong

**Keywords:** prosocial behaviour, individualism, future orientation, identity, Chinese youth

## Abstract

There is a lack of research directly examining the relationships between future orientation, individualism, prosocial engagement and identity status among Chinese youth. This study focuses on the moderating role of identity status in the relationship between individualistic values, future orientation and prosocial behaviours. The study sample consists of 1817 Chinese youth aged between 15 and 28. Six patterns of identity statuses were identified by a hierarchical cluster analysis. Path analysis was conducted to examine the relationship between the independent variables and youths’ prosocial engagement and the moderating effects of identity status. The results showed that future orientation is significantly related to prosocial engagement, while individualistic value is not significantly associated with it. The interaction of future orientation and identity status significantly affects prosocial engagement. The effect of future orientation is greater for those in searching moratorium and carefree diffusion and lower for those in achievement and foreclosure. These imply that time perspective intervention may facilitate the prosocial engagement of students who lack a mature and committed identity.

## 1. Prosocial Engagement of Youth

Prosocial engagement encompasses actions like volunteering, cooperation and charitable giving (McDougle & Li, 2023; Thielmann et al., 2020), benefiting both individuals and society by promoting physical, informational, and emotional support (Eisenberg, 1986; Siu et al., 2012). These behaviours can be informal or formal, and they may involve unpaid services in emergencies or continuous situations (Finkelstein & Brannick, 2007). In addition to the benefits to others, prosocial engagement offers significant advantages for young people. It is associated with lower levels of externalizing behaviour, such as rule-breaking and aggressive behaviour, and fewer internalizing psychological symptoms, including depression and loneliness (Flynn et al., 2015). Furthermore, prosocial engagement helps young people fulfil their personal needs and enhances their self-confidence, self-awareness, role functioning, and interpersonal relationships (C. E. Schwartz et al., 2009; Weinstein & Ryan, 2010; Wentzel & McNamara, 1999). While previous studies have primarily focused on the outcomes of prosocial engagement, the factors driving these behaviours differ across cultural and social contexts.

Research has shown that demographic backgrounds can influence prosocial engagement. Sex, age, and education level are strongly related to prosocial behavioural tendencies. Female and older Greek young adults tend to be more prosocial than those who are male and younger (Lampridis & Papastylianou, 2017). Females have stronger prosocial behavioural tendencies due to gender role socialization. Older youth are more aware of the benefits of prosocial behaviour. Education is strongly associated with prosocial engagement because of greater awareness of social problems and empathy towards others (Penner et al., 2005). High parental education also promotes the prosocial behaviour of children by offering better parenting and involvement which generate more social capital (Siu et al., 2012). Psychologists consider personal traits to be antecedents to individual differences in prosocial behaviour (Thielmann et al., 2020). In a meta-analysis of Thielmann et al. (2020), social value orientation was found to be one of the traits with the strongest relationship with prosocial behaviour, highlighting its role in explaining individual differences in this behaviour (Penner et al., 2005). A theoretical framework of individual differences in prosocial engagement based on personality traits was proposed by Thielmann et al. (2020). This study adopts this framework by focusing on how social values and identity status affect the prosocial behaviours of Chinese youth. Individualism and future orientation are considered two important values in late-modern societies, and identity status is the moderator of the effects of these values on prosocial behaviours.

Chinese youth are influenced by family-oriented and collectivist values that emphasize community support, contrasting with the individual-focused prosocial tendencies seen in Western cultures (Main et al., 2017). They place a greater emphasis on assisting their families compared to European youth, which motivates them to help others within their communities (Fuligni et al., 1999). A study in Hong Kong found that socialization and support from peers, schools, and parents are essential for developing prosocial intentions (Lai et al., 2015). These social factors play a significant role, while individual factors like empathy and moral reasoning are less impactful. However, some studies from abroad emphasize that individual factors, such as motivations and personal satisfaction, are crucial in predicting prosocial behaviour (Chacón et al., 2007; Eisenberg et al., 2010; Finkelstein, 2008; Laible et al., 2014). As global culture increasingly influences Chinese society, the traditional emphasis on social responsibilities may change. Further research is needed to understand the impact of personal and social factors on youth behaviour in Hong Kong, particularly regarding the growing individualistic values and their potential to enhance prosocial engagement among Chinese youth.

Recent Chinese studies have explored the relationship between identity and prosocial engagement. The results have shown that in-group identity and moral identity moderate or mediate the effect of perceived social support on the prosocial engagement of children and college students (Li & Hu, 2023; Xiong et al., 2021). Also, moral identity has been found to promote prosocial engagement among Chinese undergraduate and graduate students (Ding et al., 2018). Those with a strong sense of moral identity are more inclined to participate in prosocial activities driven by virtues such as generosity (Septianto & Soegianto, 2017). Community identity also serves as a mediator between socioeconomic status and prosocial engagement (Wang et al., 2021). Strengthening one’s prosocial role identity effectively facilitates prosocial engagement, as it aligns with role expectations (McDougle & Li, 2023). While group and moral identity have been extensively studied, future research could explore the relationship between prosocial engagement and identity formation (Flynn et al., 2015).

### 1.1. Individualistic Value and Prosocial Behaviour

Prosocial engagement is related to personal cultural tendencies. Individuality is a significant value in late-modern society. Individuals who embrace individualistic values tend to have a sense of independence and uniqueness. They determine their behaviour based on personal attitudes, focus on self-enhancement and pursue personal rather than social goals (Cozma, 2011; Hui, 1988; Triandis, 2001). Individualists are primarily concerned with their own interests, whereas collectivists show a greater willingness to contribute to their community. Collectivists value group membership, prioritize group goals, define their roles and responsibilities through their relationships with others, and adhere to group expectations to maintain harmonious relationships (Cozma, 2011; Hardy et al., 2010). This collectivistic orientation is positively related to altruistic motivations and the development of the volunteer role identity (Finkelstein, 2010). High levels of social cohesion are associated with more generalised trust and prosocial behaviour (van den Bos et al., 2018). Previous research suggests that social cohesion facilitates cooperative behaviour, while social norms motivate individuals to initiate prosocial behaviours to foster positive social relationships. Individuals with strong collectivist values are likely to engage in prosocial behaviour. Those with strong collectivist values are more likely to engage in prosocial behaviours, as they feel a responsibility to help members of their in-group (Triandis, 2001). Conversely, individualists may perceive less social responsibilities and are less likely to serve their communities. Social value orientations can differ across cultures as horizontal or vertical. According to Triandis and Gelfand (1998), people in vertical cultural settings prioritize hierarchy, while those in horizontal cultural settings prioritize equality. People with individualistic values prioritize their own independence and personal goals over community interests. They can be categorized into two groups: horizontal and vertical individualists (Triandis, 2001). Horizontal individualists see everyone as equal and independent (Singelis et al., 1995), while vertical individualists focus on competition and personal success. Research shows that prosocial individuals support fewer vertical individualistic values and more horizontal collectivistic values (Lampridis & Papastylianou, 2017). These studies suggest that people with strong vertical individualistic values are less likely to engage in prosocial or civic activities, while those with horizontal values do not significantly affect their prosocial engagement. However, there is limited research on how individualistic values impact people in societies that emphasize collectivism.

Under the influence of collective values, the influence of individualist values on the Chinese may differ from those of the Westerners. Although younger Chinese generations endorse individualistic values, traditional Confucian relationships and family values remain fundamental in Chinese societies and have stronger effects on them than individualistic values (Chen, 2015; Weng et al., 2021). Offering support to family members and a sense of obligation towards the family are core in the Chinese Confucian culture. The transmission of traditional familism values can foster prosocial behavioural tendencies (Calderón-Tena et al., 2011). Familism values promote compliant, emotional and dire prosocial behaviours (Zhao et al., 2022). Prosocial practices in the family, such as providing caretaking and household responsibilities, promote more generalized prosocial behaviours toward others. The influence of collectivistic values may minimize the negative effects of individualistic values. Nevertheless, younger Chinese generations emphasise independence from their families of origin and develop individualistic and collectivistic traits due to the influence of Western cultural products (Chen, 2015). Western and Chinese values are both endorsed by Hong Kong Chinese youths and adolescents and are integrated into their meaning system (Chiu & Fu, 2001; Wang et al., 2021). Chinese culture mainly affects their moral values, while Western culture mainly affects their achievement values (Chiu & Fu, 2001). The value systems that emphasize interdependence and independence work together to help shape their bicultural self (Wang et al., 2021). Pragmatic individualistic values, such as self-centeredness and materialism, are negatively related to the prosocial engagement of Chinese high school students in Hong Kong (Siu et al., 2012). The present study will explore the effects of the two dimensions of individualism on prosocial behaviour separately to examine the role of individualist values in affecting prosocial actions in the late-modern Chinese context.

### 1.2. Future Orientation and Prosocial Behaviour

Individuals who focus on the future are more likely to initiate and sustain prosocial behaviours (Maki et al., 2016). They consider how their actions can affect their future well-being and that of others. This consideration of consequences can motivate them to engage in prosocial activities, leading to greater satisfaction from their contribution and increased involvement. Many prosocial actions, such as volunteering, are planned activities that involve consideration for the future. Future-oriented individuals are more inclined to make plans, including those for volunteering (Zimbardo & Boyd, 1999), and are dedicated to achieving long-term outcomes. People who are aware of future consequences are more willing to invest time and effort to achieve desirable outcomes for the local community (Trommsdorff, 1986). Community development can benefit both society and the individuals engaged in it. Future-oriented individuals may possess a strong sense of social responsibility, recognizing that their present actions can shape the future for themselves and others. Their prosocial behaviour reflects these responsibilities and a desire to contribute to a better community. They may participate in prosocial activities as a means of fulfilling their social obligations and making a positive impact on society. However, there is currently a lack of recent studies exploring the relationship between future orientation and prosocial behaviours.

Prosocial behaviour aligns well with the goals and aspirations of future-oriented individuals who understand the positive impact it can have on their relationships, reputation, or personal growth. These individuals may engage in prosocial behaviour to achieve their long-term objectives. Although prosocial behaviour sometimes requires sacrificing immediate self-interest for the benefit of others, future-oriented individuals are often more willing to take part in such actions, even when they do not yield immediate rewards. They may delay immediate gratification because they recognize the potential long-term benefits for themselves or society (Trommsdorff, 1986). Future-oriented individuals typically value and invest in long-term relationships, understanding that maintaining positive social connections is important for their future well-being (Rydell & Brocki, 2024). Engaging in prosocial behaviour can strengthen social bonds and contribute to developing a supportive network. Consequently, future-oriented individuals may engage in prosocial activities to build and maintain relationships that they believe will be beneficial in the long run. While existing studies do not focus directly on the relationship between future orientation and prosocial engagement, a more comprehensive analysis of this relationship is necessary. The present study aims to investigate whether youths with higher levels of future orientation are more likely to engage in prosocial behaviour.

### 1.3. Youth Identity and Prosocial Behaviour

Research suggests that youth with well-developed identities tend to engage in more prosocial behaviours. Identity formation is an important developmental task during adolescence and emerging adulthood as it significantly impacts various aspects of adult life, such as job choices and sexual partners (Crocetti et al., 2014). Adolescents often shape their self-concept through identity with other groups (Bruner et al., 2018). The process of youth identity development is a dynamic process that is characterized by a tension between synthesis and confusion. A synthesized identity is linked to positive well-being, a strong self-image, and higher levels of prosocial behaviour. In contrast, youths with a confused identity are more likely to exhibit negative emotions and behaviours, such as internalizing symptoms (e.g., depression and anxiety) and externalizing symptoms (e.g., substance abuse and physical aggression). Prosocial engagement can empower and support young people’s identity development (Marta & Pozzi, 2008). Those who view prosocial engagement as an opportunity to learn and explore their capabilities are often more motivated to join these activities. Scholars have examined the relationship of prosocial and civic engagement with different aspects of identity. Young people with mature identity development tend to be more active in community activities and more inclined to help others, while those with less developed identities are typically less involved (Crocetti et al., 2012; Hardy et al., 2010; Pancer et al., 2007). Achieving a mature identity is associated with more positive social attitudes and stronger moral motivations, leading these youths to become active citizens. They often develop a strong sense of social responsibility and a desire to contribute to their communities (Crocetti et al., 2012). A stable identity fosters their inclination to participate in society through various community activities, and achieving a mature identity can also motivate further community involvement (Hardy et al., 2010). Community involvement plays a vital role in identity formation, facilitating positive development among youth. Engaging in social activities provides opportunities for young people to exercise their agency and strengthen their sense of self-efficacy and social connectedness (Hardy et al., 2010).

Based on Marcia’s (1993) conceptualisation of identity status, Luyckx et al. (2010) developed a six-status model based on five dimensions: commitment making, identification with commitment, exploration in breadth, exploration in-depth, and ruminative exploration. Young people with Achievement status have high levels of all identity dimensions except for ruminative exploration. Those with Foreclosure status have a high level on both commitment dimensions but low on the exploration dimensions. Those with Moratorium status are in the transitional stage and have low levels of both commitment dimensions and high levels of exploration in breadth and depth, accompanied by an average to a high level of ruminative exploration. Those with Searching Moratorium status have a moderate to high level on both commitment dimensions and high on exploration dimensions. Young people with Carefree Diffusion and Diffused Diffusion have a low level on both commitment dimensions and are from low to average on exploration in breadth and depth. However, they are different regarding ruminative exploration. Young people with Diffused Diffusion have a high level of ruminative exploration, while those with carefree diffusion have a low level of ruminative exploration. The present study focuses on the influence of identity status on prosocial engagement. Young people with Achievement, Searching Moratorium, and Moratorium statuses are expected to have a high level of prosocial engagement because they actively evaluate and have a high intention to explore various identity alternatives. In addition, prosocial engagement offers multiple opportunities for these young people to explore themselves. Therefore, it is anticipated that the high exploration of young people with Achievement, Searching Moratorium, or Moratorium statuses will facilitate the positive effects of future orientation while reducing the adverse impact of individualistic values on their prosocial engagement.

### 1.4. The Present Study

The rise of individualistic values in the context of globalisation may undermine the sense of social responsibility, promote individual autonomy, and affect prosocial behaviour. In collectivist societies like China, young people may also be influenced by individualist values. If this study confirms the extent of this influence, it will allow for appropriate action to be taken to shift these values in a way that encourages prosocial behaviour. The present study specifically focuses on the Chinese youth in Hong Kong, a group facing significant changes and challenges in their lives within late-modern society. During emerging adulthood, individuals encounter numerous opportunities while navigating an uncertain future that necessitates independent exploration of love, education, work and values (Arnett, 2000). All these aspects are related to identity exploration and development, affecting their behaviour. We expect this study’s results will help practitioners understand the relationships between individualistic values, future orientation, identity status and prosocial behaviours. Additionally, we aim to confirm the role of mature identity in facilitating prosocial behaviours. Previous interventions have effectively fostered a future-focused mindset (Maki et al., 2016). Once the relationship between future orientation and prosocial behaviours is established, practitioners will be advised to implement time perspective interventions for their young clients to foster positive future orientation (Zimbardo & Sword, 2017). Overall, it is anticipated that individualistic values and future orientation will serve as predictors of prosocial behaviour, with identity status as a control variable that influences the relationship between individualistic values and future orientation and prosocial behaviour.

Prosocial tendency is related to rational decision-making and is influenced by motivation, resource availability, and social values (Cardona-Isaza et al., 2023). According to the cost–benefit framework (Contreras-Huerta, 2023), prosocial behaviours are actions that benefit others at the individual’s own cost. The perception of cost and benefits is subjective; individuals who are sensitive to other’s welfare are more likely to engage in prosocial behaviours even with minimal benefits regardless of the costs involved. In contrast, those who are more aware of the cost may hesitate to act prosocially, even when the costs are low and potential benefits are high (Contreras-Huerta, 2023). Based on this cost–benefit framework, this study posits that individualistic values and future orientations are antecedents to prosocial behaviours, with identity status as a moderator between these variables. Youth with individualistic values tend to focus on personal costs and are less likely to engage in prosocial behaviours. Conversely, those with future orientation are willing to sacrifice immediate costs for potential long-term benefits by participating in prosocial actions. While previous studies have suggested a relationship between future orientation, individualistic values and prosocial behaviours, none has explored the moderating role of identity status in this relationship.

An individual’s identity status can significantly influence the relationship between future orientation and prosocial behaviour. People with a mature identity—characterized by a clear understanding of their values, beliefs, and life goals—are more inclined to think about the future and engage in planning (Zimbardo & Boyd, 1999). This self-awareness motivates them to act in ways that align with their values and goals, leading to more prosocial behaviour. A mature identity is an internal mechanism that transforms future-oriented intentions into concrete actions that benefit others. Individuals with mature identities typically possess a stronger sense of personal responsibility and commitment to others, which helps translate future-oriented thinking into actual prosocial behaviours (den Boer et al., 2021). In contrast, an immature identity status can weaken the relationship between future orientation and prosocial behaviour. When individuals lack a clear or consistent sense of self, they often struggle to establish meaningful goals and plan for the future. This lack of clarity can hinder their ability to engage in prosocial behaviours that require future-oriented thinking and planning. As a result, they may be more prone to seek immediate gratification rather than considering long-term implications.

Mature identity also plays a significant role in moderating the relationship between individualism and prosocial behaviour. It can help mitigate the adverse effects of individualistic values on prosocial actions. Young people who embrace individualistic values often prioritize personal freedom and goals over the well-being of others. In contrast, those with a mature identity possess a better understanding of their values and social responsibilities, recognizing the importance of considering others’ needs and engaging in prosocial behaviours (Piotrowski et al., 2020). Consequently, individuals with a mature identity are more likely to demonstrate prosocial behaviour, even if they hold individualistic values. Additionally, a mature identity can serve as an internal mechanism that translates individualistic values and goals into prosocial actions. Youths with a mature identity recognize individual autonomy while valuing the community’s well-being. This balance allows them to pursue their personal goals while also engaging in acts of compassion or assistance that reflect their broader sense of self.

The present study addresses the research gap by conducting a more thorough analysis of the association between individualistic values, future orientations, and prosocial behaviours. It also seeks to investigate whether identity status can significantly moderate this relationship. Based on the above overseas literature, this study develops the following hypotheses.

1. Individualistic value is negatively related to prosocial behaviour, while future orientation is positively related to it;
2. Identity status moderates the relationship between individualistic values and prosocial behaviours as well as the relationship between future orientation and prosocial behaviours.

## 2. Procedure and Methods

The data came from the first phase of a longitudinal study conducted by the authors about youth identity status and psychosocial well-being. Participants were recruited from 14 secondary schools and 36 higher educational institutions in Hong Kong. Secondary schools were selected randomly from the school lists in Hong Kong, and invitation letters were sent to the selected schools for consent to distribute the questionnaire to their students. This study included secondary schools and higher education institutions from various districts and different banding systems. The research assistant contacted the students onsite or online to collect the data. College students were invited through the researchers’ networks. A professional survey company assisted with data collection. The company is a research institution established in 2000 by a university in Hong Kong, and it became independent in 2009. It has conducted more than 600 research projects for academic staff of universities and government units in Hong Kong. Invalid cases were removed from the sample. Participants who completed the questionnaire in less than 10 min and showed repeated patterns in scales (especially with reversed items) were considered as providing invalid responses. A total of 2333 samples were collected, of which 493 were excluded based on the abovementioned criteria. Additionally, 23 cases were removed for not meeting the cluster-analysis criteria. Therefore, the final sample size is 1817. Information was successfully obtained from 1817 youth aged between 15 and 28. There were 727 male respondents and 1090 female respondents. The demographic information of participants is stated in Table 1. The mean age was 18.94 with a standard deviation of 2.96. This study involved 958 secondary school students and 882 college students. Of these, 1495 were local-born Hong Kong residents, and 322 were non-local-born residents. The non-local-born students came from Mainland China and Macau and have similar cultural backgrounds to those of local Hong Kong Chinese students. A total of 29.0% of the respondents’ fathers had attained a high school education, while 14.4% had a college education or higher. Meanwhile, 34.1% of the respondents’ mothers had completed high school, while 12.2% had a college education or higher. A total of 49.8% of the respondents came from government-aided secondary schools, 2.4% from government-owned or direct subsided secondary schools, 32.8% from self-financed degree programmes, 10.2% from government-funded degree programmes, 4.4% from self-financed sub-degree programmes, and 0.4% from government-funded sub-degree programmes. Among the respondents, 443 (24.22%) were classified as Moratorium; 413 (22.73%) were Searching Moratorium; 391 (21.52%) were Foreclosure; 236 (12.99%) were Carefree Diffusion; 188 (10.35%) were Achievement and 146 (8.04%) were Diffused Diffusion.

### 2.1. Ethical Approval

Online informed consent was obtained from the participants. Participants were required to indicate their acceptance to join this study before starting to fill in the online questionnaire. Parents of participants under 18 years old from secondary schools were also informed via email or letter. Ethical approval for this study was obtained from the University’s research ethics committee. No animals were used in this research. All human research procedures followed were in accordance with the ethical standards of the institutional committee and with the Declaration of Helsinki.

### 2.2. Measures

#### 2.2.1. Prosocial Engagement

The 16-item Prosocialness Scale for Adults was adopted to assess four fundamental aspects of prosocial behaviours: helping behaviours, sharing, attempting to take care of others, willingness to help and assist the needy, and feeling empathic with others (Caprara et al., 2005). Participants were asked to rate the extent of each item in describing themselves on a 5-point Likert scale, ranging from 1, never true, to 5, almost always true. Examples of scale items contain statements such as, “I share the things that I have with my friends” and “I try to be close to and take care of those who are in need”. Statements include helping behaviours toward both in-group and out-group members. The higher the score, the greater the level of prosocial behaviour. The scale has confirmed adequate psychometric properties, good reliability, and high construct validity (Caprara et al., 2005). The Cronbach’s Alpha of this scale in this study is 0.92, which implies very good reliability.

#### 2.2.2. Individualistic Value

The 8-item short-form measurement of individualism (IND) was used (Triandis & Gelfand, 1998). The scale has been proven to have configural and metric equivalence, good reliability, as well as good convergent and divergent validity (Cozma, 2011). The construct of IND comprises two factors, horizontal individualism and vertical individualism, each with four items. Examples of scale items contain statements such as, “I’d rather depend on myself than others” and “Competition it the law of nature”. The Cronbach’s Alpha of horizontal individualism and vertical individualism sub-scales in this study are 0.81 and 0.80, respectively, which implies good reliability.

#### 2.2.3. Future Orientation

Five items from the Consideration of Future Consequences Scale were employed to measure future orientation (Strathman et al., 1994). Respondents indicated whether or not the statement is characteristic of themselves from a 5-point Likert Scale from 1, “extremely uncharacteristic”, to 5, “extremely characteristic.” Examples of scale items contain statements such as “Often I engage in a particular behaviour in order to achieve outcomes that may not result for many years” and “I am willing to sacrifice my immediate happiness or well-being in order to achieve future outcomes”. The one-dimension scale has been confirmed with very good internal consistency in four samples of university students with a Cronbach’s alpha between 0.80 and 0.86 (Strathman & Joireman, 2005). The convergent validity of the scale was supported by its relationship with other individual-differences measures (Strathman et al., 1994). The Cronbach’s Alpha of this scale in this study is 0.78, which implies good reliability.

#### 2.2.4. Identity Status

The 25-item Dimensions of Identity Development Scale (DIDS) was employed to measure the identity status of participants. The scale is rated on a 5-point Likert Scale ranging from 1 (strongly disagree) to 5 (strongly agree), which consists of five items per each of the five identity dimensions: commitment making, identification with commitment, exploration in breadth, exploration in depth, and ruminative exploration (Luyckx et al., 2008). Examples of scale items contain statements such as “I have decided on the direction I am going to follow in my life”, “I think about different goals that I might pursue”, “I worry about what I want to do with my future”, “My future plans give me self-confidence” and “I think about the future plans I already made”. A mean score was computed for each dimension; the higher the score, the greater the level. Participants were classified into six identity clusters based on the mean score of these dimensions. The five identity dimensions model was proved to have good internal and external validity (Luyckx et al., 2008).

Using IBM Statistics SPSS (version 27), cluster analyses were conducted for each sample using a two-step procedure (Gore, 2000). Before the implementation of this clustering procedure, 18 multivariate outliers (with high Mahalanobis distance values) were removed. Firstly, a hierarchical cluster analysis was conducted on the five identity dimensions using Ward’s method based on squared Euclidean distances. After evaluating the four- to six-cluster solution, a 6-cluster solution was retained based on the theoretical interpretability, parsimony, and explanatory power of the cluster solution (the cluster solution had to explain at least 50% of the variance in the different identity dimensions). Secondly, the initial cluster centres were used in an iterative *k*-means clustering procedure. As a result, six patterns of identity statuses are identified from the scale: Carefree Diffusion, Moratorium, Achievement, Diffused Diffusion, Searching Moratorium, and Foreclosure. The five sub-scales of DIDS have good reliability (Commitment making = 0.89, Identification with commitment = 0.85, Exploration in breadth = 0.84, Exploration in depth = 0.77, and Ruminative exploration = 0.80). A confirmatory factor analysis was conducted to examine the factor structure of DIDS. The results confirmed the six patterns of identity statuses with satisfactory to good model fit (RMSEA < 0.001, CFI = 0.918, TLI = 0.901 and SRMR = 0.064).

Similar to the findings by Luyckx et al. (2008), the Achievement cluster scored high on both commitment dimensions and exploration in depth, intermediate on exploration in breadth, and from low to very low on ruminative exploration. These youths have high commitment and exploration with mature identity status. They continue to gather information and strongly identify with their choices. The Foreclosure cluster scored moderately high on commitment and moderately low on all exploration dimensions. These youths commit to an identity and values that others place on them without exploring. Moratorium was characterised by a low score on both commitment dimensions and a from moderate to high score on exploration dimensions. The Moratorium status represents the transitional crisis. These youths continue to search for alternatives but fail to identify with any option and feel pressured to make a specific choice. The Searching Moratorium cluster scored moderately high in all dimensions. Youths with searching moratorium status are more adaptive than those with Moratorium status who are unsuccessful in finding alternative commitments (Meeus et al., 2012). In contrast, participants in the Diffused Diffusion cluster scored from low to very low on commitment and exploration dimensions and high on ruminative exploration. Diffused Diffusion represents individuals who have attempted to develop a sense of identity but seem to be locked in a ruminative cycle of perpetual exploration (S. J. Schwartz et al., 2012). Carefree Diffusion was characterised by from low to intermediate scores on both commitment dimensions, very low scores on exploration in breadth and depth, and moderately low scores on ruminative exploration. These youths lack an interest in engaging in any form of exploration and are unbothered by the absence of commitment (Luyckx et al., 2011).

The researchers translated all scales from English to Chinese and then back-translated them to the original version, consulting a native English scholar for appropriateness. Before the data collection, a pilot test with 465 samples was conducted. Based on the results, the Chinese wordings of the scales were further revised, and all scales showed good internal consistency.

### 2.3. Analytical Plan

First, the three independent variables were mean-centred to obtain a Z-score for analysis. Given the moderator, identity status is a six-class categorical variable; five dummy variables were created and then interacted with three independent variables to obtain 15 interactions in SPSS 27.0. After that, two models were run to conduct path analysis in Mplus 7.4; the first model contains only the independent variables (future orientation, horizontal individualism and vertical individualism) to determine whether the three independent variables accounted for a significant amount of variance in youths’ prosocial engagement. Next, the interaction terms for identity status were added in the second model. The Searching Moratorium group was chosen as a reference group because these young people are highly explorative but have not yet developed a mature identity status. Gender, age, and education level were added as control variables in the two models.

## 3. Results

Descriptive statistics and correlations among independent and dependent variables are stated in Table 2. The mean score of prosocialness is 3.58 out of 5, implying respondents have moderate levels of prosocial engagement. The mean score of future orientation is 4.59 out of 7, which implies moderate levels of consideration of future consequences among participants. The mean scores of horizontal individualism and vertical individualism are 6.31 and 5.84 out of 9, respectively, implying that young Chinese people in Hong Kong value independence and equality among individuals, but they place less emphasis on competition and personal advancement. According to the correlational analyses, prosocial engagement is positively related to future orientation, horizontal individualism, and vertical individualism (β = 0.34, *p* < 0.001; β = 0.09, *p* < 0.001; β = 0.08, *p* < 0.001). Future orientation is positively related to horizontal individualism and vertical individualism (β = 0.30, *p* < 0.001; β = 0.19, *p* < 0.001). Horizontal individualism and vertical individualism are positively related (β = 0.41, *p* < 0.001).

In model one, after controlling gender, age, local/nonlocal, father/mother education levels, and participant’s education level, the results indicated that future orientation is significantly and positively related to prosocial engagement (*b* = 0.34, *p* = 0.000), but two dimensions of individualism are both insignificantly related to prosocial engagement (horizontal individualism, *b* = −0.03, *p* = 0.216; vertical individualism, *b* = 0.02, *p* = 0.390). Respondents with an identity status with a high level of exploration in breadth and exploration in depth have a high level of prosocial engagement, such as achievement (*b* = 0.67, *p* < 0.001), moratorium (*b* = 0.44, *p* < 0.001), and searching moratorium (*b* = 0.60, *p* < 0.001). Consistent with previous studies, females have more prosocial behaviour than males (*b* = 0.21, *p* < 0.001). Non-local students have more prosocial behaviour than local students (*b* = −0.08, *p* < 0.001). However, age, education levels of the father and mother, and participant’s education level are unrelated to prosocial engagement (*b* = −0.01, *p* = 0.580; *b* = 0.01, *p* = 0.692; *b* = −0.01, *p* = 0.593; *b* = −0.06, *p* = 0.467). The findings of this study are at odds with previous research suggesting that age and education influence prosocial behaviour. The specific educational context in Hong Kong may diminish the effects of these factors. In Hong Kong, senior secondary students are required to participate in well-organized community service programs integrated into the school curriculum. This involvement facilitates high school students’ engagement in prosocial behaviours. As a result, younger secondary students in Hong Kong may demonstrate levels of prosocial engagement comparable to, or even higher than, those of older college students.

In model two, the moderation analysis indicated that the relationship between future orientation and prosocial engagement in the Diffused Diffusion and Foreclosure groups was significantly different from the relationship between future orientation and prosocial engagement in the Searching Moratorium group (Diffused diffusion, *b_interaction_* = −0.28, *p* = 0.006; Foreclosure, *b_interaction_* = −0.16, *p* = 0.036). In these three groups, future orientation is significantly related to prosocial engagement, except for in the Diffused Diffusion group (Searching Moratorium group: *b* = 0.38, *p* = 0.000; Diffused Diffusion group: *b* = 0.10, *p* = 0.271; Foreclosure group: *b* = 0.22, *p* = 0.000). The results indicated that future orientation had a stronger positive relationship with prosocial engagement in the Searching Moratorium group than in the Diffused Diffusion and Foreclosure groups.

**Figure 1:**
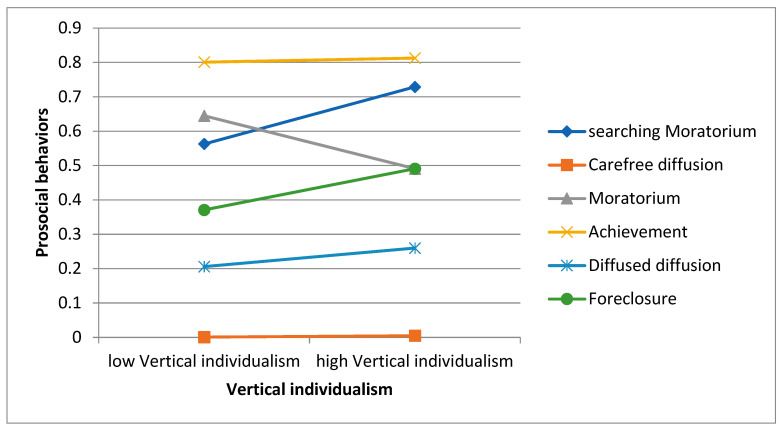
Results for the moderating role of identity in the relationship between vertical individualism and prosocialness (*N* = 1817).

For individualism, the moderation analysis indicated that the relationship between vertical individualism and prosocial engagement in the Moratorium group is significantly different from the relationship between vertical individualism and prosocial engagement in the Searching Moratorium group (Moratorium, *b_interaction_* = −0.16, *p* = 0.021). In the Searching Moratorium group, vertical individualism is insignificantly related to prosocial engagement (Searching Moratorium group: *b* = 0.08, *p* = 0.105). In the Moratorium group, vertical individualism is related to prosocial engagement at a marginally significant level (*b* = −0.08, *p* = 0.098). The results indicated that vertical individualism is more likely to have a negative association with prosocial behaviours in the Moratorium group compared with the reference group (see Figure 1).

## 4. Conclusions and Discussion

The results of the path analysis indicated that youth who have a mature identity, characterized by high levels of both breadth and depth of exploration, engage in more prosocial behaviours compared to those with lower levels of exploration in both areas. Openness and curiosity are vital to identity exploration (Luyckx et al., 2008). Prosocial engagement is one of the means that allows youth to explore their goals, values, and beliefs before making commitments. Therefore, youth with Achievement, Moratorium, and Searching Moratorium statuses have more prosocial engagement. This finding implies that having a more mature and adaptive identity status may result in greater prosocial engagement. The continuous search for various alternatives and attitudes is essential for encouraging prosocial actions. However, youth with a Foreclosure identity show high commitment but low exploration, which discourages prosocial engagement.

The results of the present study failed to support the direct relationship between individualistic values and prosocial engagement. This study revealed that Hong Kong youth have higher levels of horizontal individualist values than vertical individualist values (*M* = 6.31/9; *M* = 5.84/9). Western values may foster independence in Hong Kong youth, but Chinese values may prevent them from prioritizing competitiveness and individual achievement. Both Western individualism and Confucianism emphasise the importance of achievement and success; Chinese youths may understand and practice them in different ways than their counterparts in Western societies (Weng et al., 2021). Although Chinese culture emphasises collectivistic values, young people in Hong Kong may be influenced by individualistic values in certain situations. According to the results of model two, identity status moderates the relationship between vertical individualism and prosocial engagement. Vertical individualism is marginally related to prosocial engagement only in the Moratorium group but is not related in other groups. Youth in the Moratorium group have low commitments but high exploration. Emphasizing individual autonomy with low commitment and high exploration may cause youth to focus on their achievement and neglect the needs of others, reducing prosocial tendencies. Other studies found that individualism is associated with career-related volunteer motivation (Finkelstein, 2010). Although individualism focuses on autonomy and self-fulfilment and favours personal goals over group goals, the autonomy treasured in individualism may encourage individuals to help strangers and become involved in civic activities to voice out and fight for their benefits. Autonomy also facilitates respect for others and a sense of personal responsibility that encourages volunteering (Waterman, 1981). The above findings contradict Triandis’s (2001) conclusion that individuals with high individualistic values are less likely to serve the community. Therefore, inconclusive results have been found in the present Hong Kong study and previous studies in other societies. Individualistic values may enhance or discourage prosocial engagement. However, the present study further suggests that vertical individualistic value is negatively related to prosocial engagement for youth with Moratorium identity status.

The limited connection between individualistic values and prosocial behaviour may stem from endorsing collectivistic values in Chinese culture. In this context, the individualistic values held by the Chinese may differ from those of Westerners. The emphasis on community and social responsibility fosters prosocial behaviour (Bartolo et al., 2023). Relational harmony and social responsibility are key components of Confucian ethics in China that encourage young people to engage in prosocial actions. These values inspire individuals to consider the needs of others, helping to counterbalance the influence of individualistic values. Among Chinese youths in Hong Kong, the coexistence of both collectivistic and individualistic values among Chinese youth may reduce the adverse effects of individualism on their engagement in prosocial activities. Further studies may be conducted to clarify the relationship between individualism and prosocial engagement in different identity statuses and compare the situations in Western and Asian societies.

**Figure 2:**
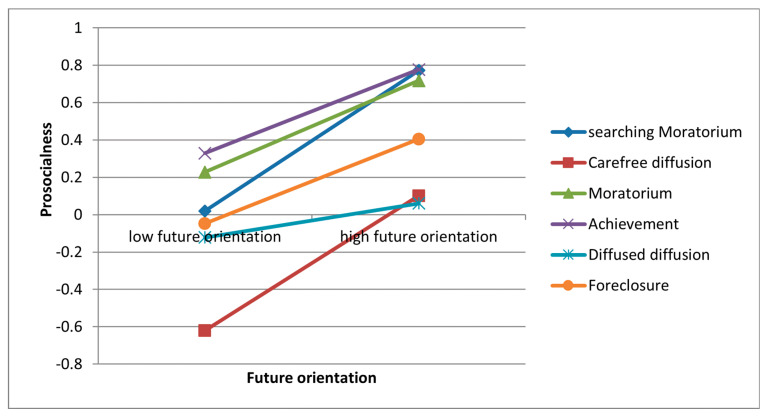
Results for the moderating role of identity in the relationship between future orientation and prosocialness (*N* = 1817).

Identity status moderates the relationship between future orientation and prosocial engagement (see Figure 2). After controlling identity status, future orientation is not related to prosocial engagement for youth with an identity status of Diffused Diffusion. In contrast, future orientation is positively associated with prosocial engagement for youth in other statuses. The relationship between future orientation with prosocial engagement is greater for those in Searching Moratorium (*b* = 0.25, *p* < 0.001) and Carefree Diffusion (*b* = 0.23, *p* < 0.001) and is lower for those in Achievement (*b* = 0.14, *p* = 0.01) and Foreclosure (*b* = 0.15, *p* < 0.001). These results imply that the relationship between future orientation and prosocial engagement for those youth who feel sure about and identify with and internalise their choices with commitment is weak. These youth with more mature identity statuses guide them on their life paths. The results above add value to the recent discussion and demonstrate that the relationship between future orientation and prosocial engagement can differ depending on a youth’s identity status.

Youth with Searching Moratorium and Carefree Diffusion have less adaptive identity with low commitment as well as from high to moderate levels of rumination and indecision (Luyckx et al., 2008). In terms of the lack of mature and committed identity, this study’s results suggest that a strong future orientation can facilitate the prosocial engagement of such individuals to further exploration. Previous studies have confirmed the positive effect of time perspective interventions on enhancing goal striving, openness to future projects, career decision-making, and the career search self-efficacy of participants (Ferrari et al., 2012; Mooney et al., 2021; Park et al., 2020). Another study also proved that time perspective intervention could enhance the regular physical activity of youth (Hall & Fong, 2003). Such interventions facilitate youth’s prosocial engagement with similar planning and goal setting. However, for those with more adaptive identity statuses, such as Achievement and Foreclosure, future orientation has less effect on them because they tend to have a clear life goal and plan for their development. Youth with Diffused Diffusion have low commitment and exploration but high rumination (Luyckx et al., 2008). Due to a lack of intention for exploration and lower levels of perspective-taking under rumination, future orientation fails to motivate the youth with Diffused Diffusion to engage in prosocial activities. Assisting these youth to develop a more mature identity may be more effective in facilitating their prosocial engagement.

Previous studies have shown that group-based identity development programs help participants discuss identity issues, enhance self-understanding, and build critical thinking and problem-solving skills (Berman et al., 2008). Using participatory and empowering approaches, these programs enable individuals to identify life challenges and find effective solutions (Meca et al., 2014). Human libraries, experience sharing, discussions about life issues, and goal setting are common teaching and learning activities covered in identity interventions. These interventions help participants set goals, create pathways to achieve them, and identify potential obstacles through self-reflection and group discussions. Digital identity interventions can also be used to incorporate social media activities, such as watching career-related videos and exploring LinkedIn profiles, to support personal identity exploration and online networking (Soh et al., 2024). Identity intervention can be used to help young people develop a more mature identity and encourage their prosocial behaviour.

This paper is based on cross-sectional data, which cannot explore the relationship between identity development and prosocial trajectory. The authors will further explore how independent variables and identity status impact prosocial behaviour, as well as the relationships among these variables, by utilizing longitudinal data from various stages of the project. Future research could examine how collectivistic values interact with individualistic values to influence the prosocial behaviour of Chinese youths. This study focuses on the relationship between identity status, individualist values, future orientation, and prosocial engagement while ignoring the influence of psychological status. Previous studies revealed that personal well-being, life satisfaction, and happiness promote prosocial engagement (González Moreno & Molero Jurado, 2024; H. Zhang & Zhao, 2021; Y. Zhang, 2022), while depression inhibits prosocial behaviour in Chinese societies (Y. Zhang, 2022). Future studies can investigate whether various psychological statuses can modify the relationship among the study variables. Moreover, information was gathered through a self-reported questionnaire, which may generate response bias. Future studies can use observation or diaries to avoid this self-reported bias. This study only focuses on Chinese youth in Hong Kong, limiting the generalizability of the findings to Chinese in mainland China and youth from other ethnicities. The results show that non-local students have more prosocial behaviours than local students, although both are Chinese. Further studies can expand the sample to youth in other regions. Nevertheless, despite the above limitations, this study offers critical insight into the relationship between individualism, future orientation, identity status, and prosocial engagement in the late-modern society that emphasises collectivistic value. This study is the first to confirm the moderating role of identity status on the relationship between future orientation, vertical individualism, and prosocial engagement. This study contributes to the recent literature by filling the gap in understanding the influence of individualism in collectivist societies. The results imply that horizontal individualism is only marginally associated with prosocial engagement, while vertical individualism is unrelated to these behaviours. The results indicate that a service aimed at enhancing the identity development of youth is recommended. Time perspective intervention can be delivered to youths with Searching Moratorium and Carefree Diffusion identity, while identity intervention can be offered to youths with Diffused Diffusion identity to facilitate their prosocial engagement.

## Figures and Tables

**Table 1:** Demographic information of participants.

	*N*	%
**Gender**		
Male	727	40.0
Female	1090	60.0
**Participant’s education level**		
Post-secondary	869	47.8
Secondary	948	52.2
**Local/non-local**		
Local	1495	82.3
Non-local	322	17.7
**Father’s education level**		
Uneducated/Kindergarten	48	2.6
Primary	201	11.1
Lower Secondary	420	23.1
Upper Secondary	504	27.7
Vocational Training	51	2.8
Matriculation	23	1.3
Post-secondary: Non-degree	74	4.1
Post-secondary: degree or above	139	7.6
Unknown	357	19.6
**Mother’s education level**		
Uneducated/Kindergarten	46	2.5
Primary	200	11.0
Lower Secondary	435	23.9
Upper Secondary	588	32.4
Vocational Training	39	2.1
Matriculation	31	1.7
Post-secondary: Non-degree	82	4.5
Post-secondary: degree or above	109	6.0
Unknown	287	15.8
**School Types**		
Government-aided secondary school	905	49.8
Government-owned or direct subsided secondary school	43	2.4
Self-financed sub-degree programmes	80	4.4
Government-funded sub-degree programmes	8	0.4
Self-financed degree programmes	596	32.8
Government-funded degree programmes	185	10.2
	Mean	SD
**Age**	18.94	2.96

**Table 2:** Mean, standard deviations and correlation matrix between variables.

Variable	1	2	3	4
1 Prosocialness	-			
2 Future Orientation	0.343 ***	-		
3 Horizontal Individualism	0.089 ***	0.299 ***	-	
4 Vertical Individualism	0.083 ***	0.193 ***	0.413 ***	-
Mean	3.58	4.59	6.31	5.84
Standard Deviation	0.64	0.95	1.42	1.56

Note: *N* = 1817; *** *p* < 0.001 with listwise deletion.

## Data Availability

The data supporting the findings of this study are available from the corresponding author upon request.
